# **Geostatistical analysis of trace elements PXRF dataset of near-surface semi-arid soils from Central Botswana**

**DOI:** 10.1016/j.dib.2016.10.010

**Published:** 2016-10-24

**Authors:** Peter N. Eze, Valiant S. Mosokomani, Theophilus K. Udeigwe, Opeoluwa F. Oyedele, Adeniyi F. Fagbamigbe

**Affiliations:** a Department of Earth & Environmental Science, Botswana International University of Science & Technology, Private Bag 016, Palapye, Botswana; b Independent Scholar, 8901 Avenue T, Lubbock, TX 79423, USA; c Department of Mathematics & Statistics, Namibia University of Science & Technology, Windhoek, Namibia; d Department of Mathematics & Statistical Sciences, Botswana International University of Science & Technology, Palapye, Botswana; e Department of Epidemiology & Medical Statistics, University of Ibadan, Nigeria

**Keywords:** Geochemistry, Botswana, Minor elements, Grid sampling, Portable X-ray fluorescence, Semi-arid soils

## Abstract

Geospatial dataset of trace elements including manganese (Mn), iron (Fe), titanium (Ti), rubidium (Rb), strontium (Sr), thorium (Th), Vanadium (V) and Zirconium (Zr) of near-surface soils in a Cu–Ni prospecting field at Airstrip North, Central Botswana were statistically analysed. Grid sampling method was used in the field data collection. The relatively new portable X-ray fluorescence spectrometer (Delta Premium, 510890, USA) technology in a “soil” mode was used to measure the concentrations of trace elements in the soils. The data presented was obtained from the average reading of two soil samples collected from same point but passed through sieves. Sequel to DOI: 10.1016/j.dib.2016.08.026 (P.N. Eze, V.S. Mokosomani, T.K. Udeigwe, O.F. Oyedele, 2016) [1].

**Specifications Table**

**Table:** 

Subject area	*Earth Sciences*
More specific subject area	*Soil geochemistry*
Type of data	*Table, figure*
How data was acquired	*Survey, grid sampling, portable x-ray fluorescence (Delta Premium, Rh Tube Anode Instrument Serial Number: 510890, USA); Fieldmaster*^®^*soil sampling sieve set*
Data format	*Raw, analyzed*
Experimental factors	*Soil samples were collected at a 30 cm depth to avoid contamination by surficial anthropogenic deposits and organic matter.*
Experimental features	*Determine the concentration levels of trace elements including Mn, Fe, Ti, Rb, Sr, Th, V, and Zr.*
Data source location	*Maibele Airstrip North, Central Botswana.*
Data accessibility	*Data is with this article*

**Value of the data**

• It can serve as a geochemical base-line data for trace element concentrations in near-surface soils developed on meta-sedimentary parent materials in semi-arid environments.
• It can be used for pedogenic modelling and simulation of trace element concentrations and redistribution.
• Since the dataset is geo-referenced, it can be used for geospatial modelling in GIS.
• Could provide a basis for arable land selection by prospective agronomists.

## 1 Data

**Fig. 1:**
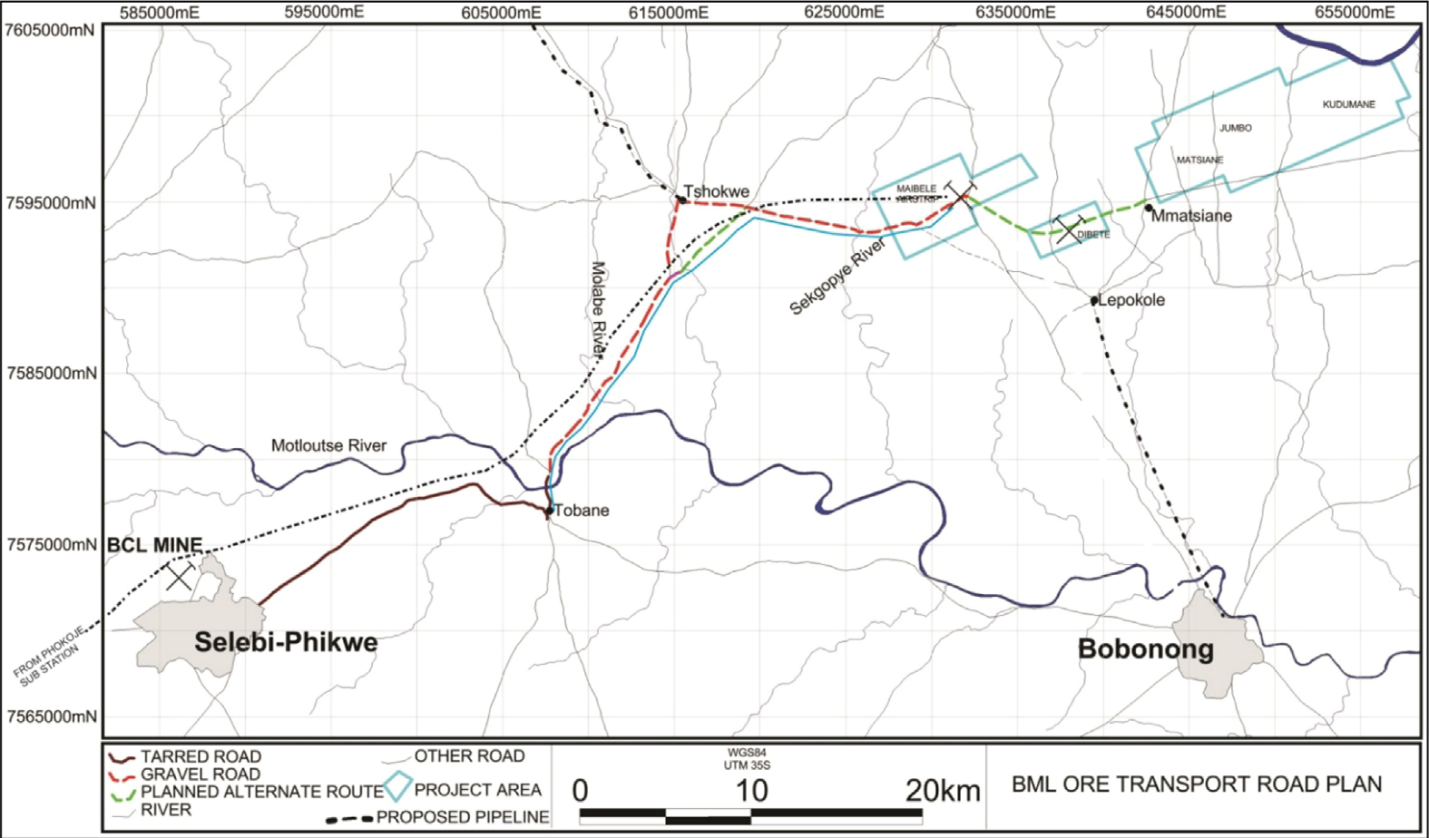
Geographical location of Maibele Airstrip North, Central Botswana.

Sequel to [1], a dataset of heavy metals, the dataset in this article consists of tables and figures which help analyze the near-surface (~30 cm depth) trace element concentrations (ppm) in soils from 1050 geo-referenced points at the Maibele Airstrip North in Central Botswana (Fig. 1) which developed on paragneisses and amphibolites parent materials [2]. The averaged reading of two samples collected from same point, their geographical coordinates and the trace elements concentrations are available in Supplementary Table 1. Portable X-ray fluorescence spectrophotometer in a “soil” mode [3] was used to determine the trace elements. The average of two readings on two samples (sieved and non-sieved) collected from the same point on the grid layout was reported.

## 2 Experimental design, materials and methods

A total of 1050 soil samples from a Cu–Ni mineral exploration field at Maibele Airstrip North were statistically analysed. To select the soils a grid method was used, where the field was first divided into 30 straight lines of equal distance apart, after which soils samples were collected at a 100 m interval along each line. The distance covered for each line/transect was about 875 m. A pit of about 30 cm depth was dug to remove organic material before collecting soil samples. Two soil samples were collected for each point, one sample was sieved using the Fieldmaster^®^ soil sampling sieve set before being placed in a labelled transparent sample bag and the other was put in a sample bag as collected (not sieved). The two samples collected at a single point were label with the same sample number but differentiate by letters at the end (for example, 1105432a and 1105432b).

A calibration standard (AMIS0329 and AMIS0316) and all soil samples were used and analysed as described in [1].

## 3 Descriptive statistics on the soil sample data

### 3.1 Bar charts

**Fig. 2:**
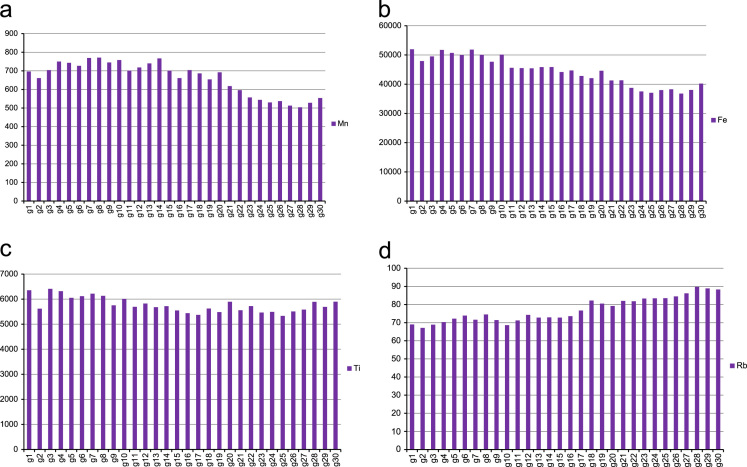

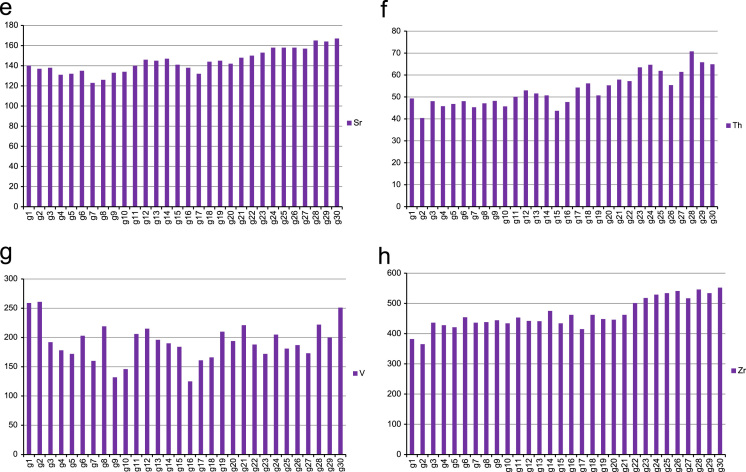
A bar chart showing the average levels of trace element a) Mn; b) Fe; c) Ti; d) Rb; e)Sr; f) Th; g) V and h) Zr per transect.

**Fig. 3:**
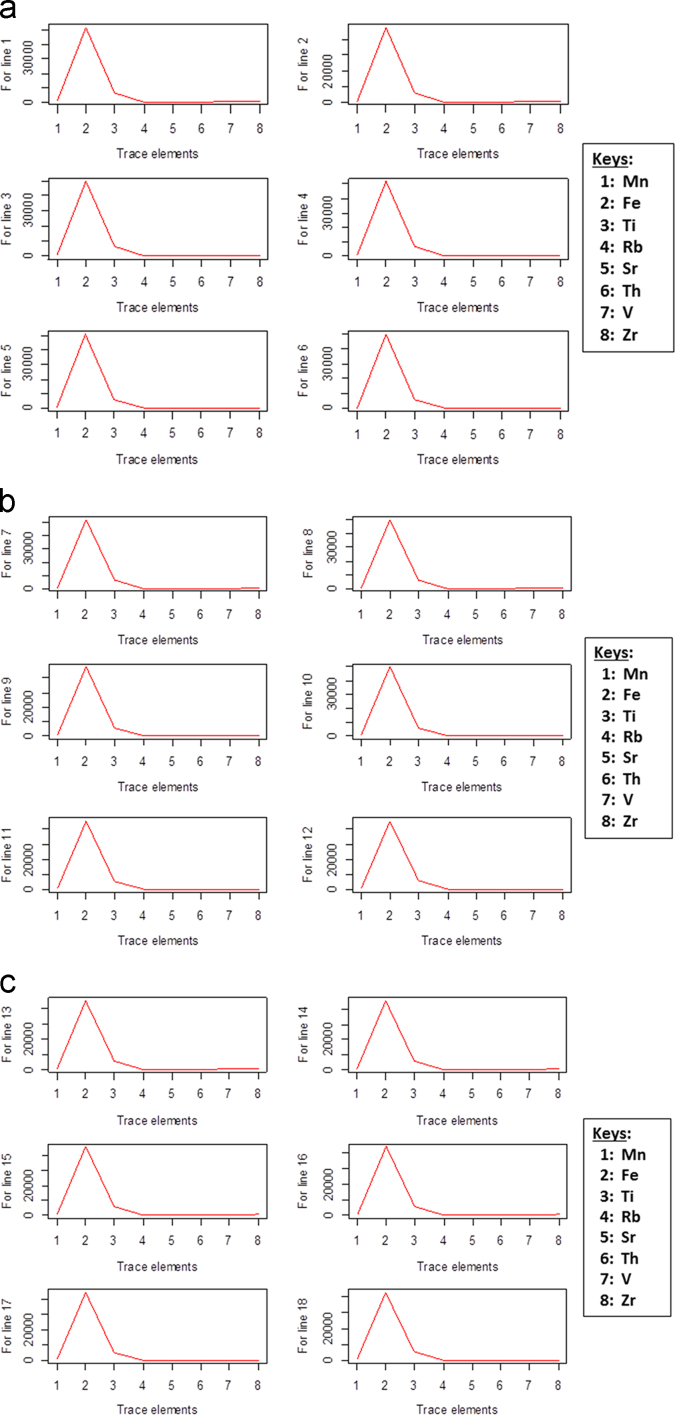

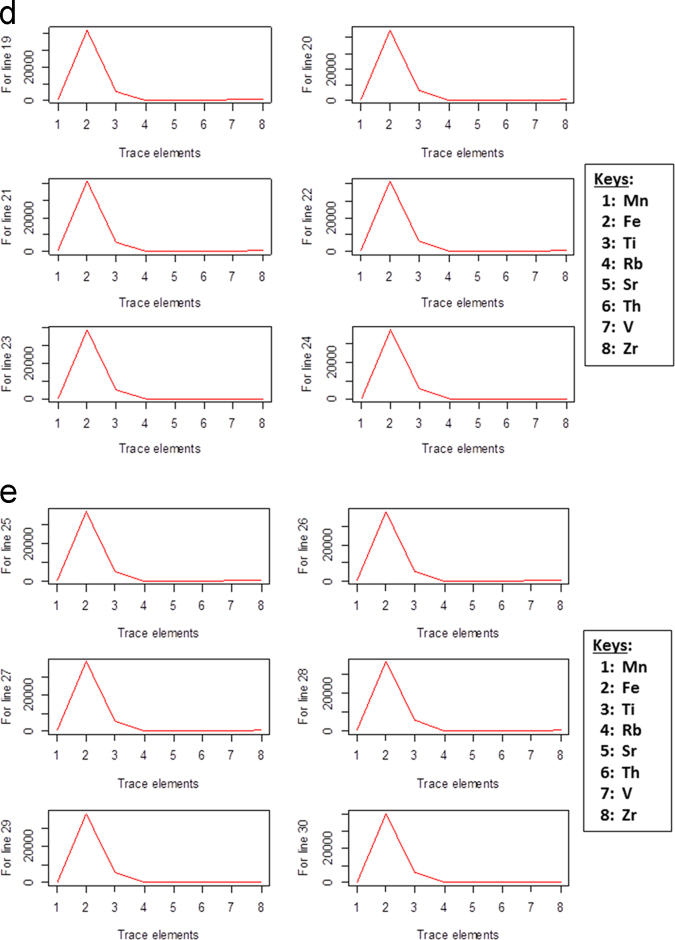
A line plot showing the average levels of trace elements for transects a) 1–6; b) 7–12; c) 13–18; d) 18–24; e) 25–30.

The bar chart of the eight trace elements from the soil sample data are shown in Fig. 2a–h and the line plots for the average levels of trace elements concentrations in Fig. 3a-e.

### 3.2 Correlation analysis on the soil sample data

The correlation values obtained from the Karl Pearson׳s correlation analysis of the soil sample data is given in Table 1 below. This table shows the estimated strength of the relationships between the eight trace elements of the soil sample data. A correlation value that is closer to (+/−) 1 can be said to have stronger relationship strength, where the (+/−) indicates the direction of the relationship − with “+” denoting positive and “−” denoting negative. A “0” value indicates that there is no correlation between the respective variables.

### 3.3 Principal component analysis on the soil sample data

#### 3.3.1 Number of factors and identifying the variables under each factor

Table 2 shows the eigenvalues obtained from the PCA of the soil sample data.

The loading values obtained from the PCA of the soil sample data is given in Table 3 below. This table shows the amount of variation that a particular variable contributed to a given factor. A variation value that is more than (+/−) 0.4 can be said to be a significant contribution to the respective factor. The contribution may vary from moderate to very high, with “0.6 and above” denoting high to very high contribution and “0.5 to 0.6” denoting moderate contribution.

## Figures and Tables

**Table 1:** Correlation values of the soil sample data.

	**Mn**	**Fe**	**Ti**	**Rb**	**Sr**	**Th**	**V**	**Zr**
**Mn**	1.000	0.938	0.648	−0.741	−0.666	−0.578	0.082	−0.699
**Fe**	0.938	1.000	0.756	−0.770	−0.707	−0.570	0.109	−0.667
**Ti**	0.648	0.756	1.000	−0.500	−0.386	−0.273	0.299	−0.268
**Rb**	−0.741	−0.770	−0.500	1.000	0.563	0.689	0.037	0.695
**Sr**	−0.666	−0.707	−0.386	0.563	1.000	0.374	0.014	0.568
**Th**	−0.578	−0.570	−0.273	0.689	0.374	1.000	0.096	0.635
**V**	0.082	0.109	0.299	0.037	0.014	0.096	1.000	0.067
**Zr**	−0.699	−0.667	−0.268	0.695	0.568	0.635	0.067	1.000

**Table 2:** Eigenvalues of the soil sample data.

**Factor 1**	**Factor 2**	**Factor 3**	**Factor 4**	**Factor 5**	**Factor 6**	**Factor 7**	**Factor 8**
**4.696**	**1.293**	0.677	0.57	0.309	0.243	0.169	0.04

**Table 3:** PCA loading values of the soil sample data.

**Trace elements**	**Factor 1**	**Factor 2**
**Mn**	**0.430**	−0.076
**Fe**	**0.440**	−0.139
**Ti**	0.309	−**0.487**
**Rb**	−**0.402**	−0.130
**Sr**	−**0.400**	−0.040
**Th**	−0.330	−**0.400**
**V**	0.027	−**0.745**
**Zr**	−**0.401**	−0.268
**Eigenvalues**	*4.696*	*1.293*
**Percentage of variance**	*58.7%*	*16.2%*
**Cumulative percentage**	*58.7%*	*74.9%*
